# Phenolic Extract from *Moringa oleifera* Leaves Inhibits Key Enzymes Linked to Erectile Dysfunction and Oxidative Stress in Rats' Penile Tissues

**DOI:** 10.1155/2015/175950

**Published:** 2015-10-05

**Authors:** Ganiyu Oboh, Adedayo O. Ademiluyi, Ayokunle O. Ademosun, Tosin A. Olasehinde, Sunday I. Oyeleye, Aline A. Boligon, Margareth L. Athayde

**Affiliations:** ^1^Functional Foods and Nutraceuticals Unit, Department of Biochemistry, Federal University of Technology, PMB 704, Akure 340001, Nigeria; ^2^Nutrition and Toxicology Division, Food Technology Department, Federal Institute of Industrial Research, Oshodi, PMB 21023, Lagos 10001, Nigeria; ^3^Phytochemical Research Laboratory, Department of Industrial Pharmacy, Federal University of Santa Maria, Building 26, Room 1115, 97105-900 Santa Maria, RS, Brazil

## Abstract

This study was designed to determine the antioxidant properties and inhibitory effects of extract from *Moringa oleifera* leaves on angiotensin-I-converting enzyme (ACE) and arginase activities *in vitro*. The extract was prepared and phenolic (total phenols and flavonoid) contents, radical (nitric oxide (NO), hydroxyl (OH)) scavenging abilities, and Fe^2+^-chelating ability were assessed. Characterization of the phenolic constituents was done via high performance liquid chromatography-diode array detection (HPLC-DAD) analysis. Furthermore, the effects of the extract on Fe^2+^-induced MDA production in rats' penile tissue homogenate as well as its action on ACE and arginase activities were also determined. The extract scavenged NO^*∗*^, OH^*∗*^, chelated Fe^2+^, and inhibited MDA production in a dose-dependent pattern with IC_50_ values of 1.36, 0.52, and 0.38 mg/mL and 194.23 *µ*g/mL, respectively. Gallic acid, chlorogenic acid, quercetin, and kaempferol were the most abundant phenolic compounds identified in the leaf extract. The extract also inhibited ACE and arginase activities in a dose-dependent pattern and their IC_50_ values were 303.03 and 159.59 *µ*g/mL, respectively. The phenolic contents, inhibition of ACE, arginase, and Fe^2+^-induced MDA production, and radical (OH^*∗*^, NO^*∗*^) scavenging and Fe^2+^-chelating abilities could be some of the possible mechanisms by which *M. oleifera* leaves could be used in the treatment and/or management of erectile dysfunction.

## 1. Introduction

Previous reports have revealed that erectile dysfunction (ED) is prevalent in over 150 million men all over the world and has been predicted to affect about 250 million men by 2025 [1]. Normal erectile function is stimulated through a series of actions involving the relaxation of cavernosal arteries and sinuses which leads to increase in blood flow to the penis [2]. These actions are mediated by nitric oxide via the activation of nitric oxide- (NO-) cyclic guanosine monophosphate (cGMP) dilator pathway and can be impaired by different factors thereby causing ED [2]. Increased arginase activity has been implicated in ED. Arginase is a metalloenzyme that converts arginine to urea and ornithine in a number of cells. There are also growing evidences that ED can be induced by high blood pressure via inveterate changes in blood pressure which can alter the flow of blood in penile vessels [3]. Moreover, angiotensin II which is obtained from angiotensin I in a reaction catalysed by angiotensin-I-converting enzyme is a potent vasoconstrictor capable of inducing vascular hypertrophy and endothelial dysfunction via decrease in the release of NO [4]. Likewise, ACE deactivates bradykinin, a vasodilator which has been implicated in erectile function via the release of NO and relaxation of corpus cavernosum [5]. The conversion of angiotensin I to angiotensin II and deactivation of bradykinin can induce high blood pressure which in turn impairs erectile function.

Oxidative stress has been linked with ED due to excessive generation of free radicals in the cavernosal tissues [6]. Superoxide combines with nitric oxide (NO) to form highly toxic peroxynitrite which is known to induce lipid peroxidation. Oxidative stress in ED reduces the availability of NO which is required for penile erection [6]. Recent trends in the management of ED involve increase in NO levels with the use of arginase inhibitors. This is because in ED there are elevated levels of arginase activity which limits NO synthase activity, reduces NO biosynthesis, and increases degradation of arginine. Antioxidants are capable of reducing oxidative stress by scavenging free radicals. Polyphenols are the most abundant antioxidants in human diet and are widespread constituents of fruits and vegetable [7]. Several studies have shown various relationships between the consumption of polyphenol or polyphenol-rich foods and diseases such as ED, diabetes, and cardiovascular and neurodegenerative diseases [8].

*Moringa oleifera *Lam. (Moringaceae) commonly known as drum stick is a medicinal plant widely grown in the tropical and subtropical regions. Reports have shown that* M. oleifera* leaves possess various pharmacological properties such as antiatherosclerosis, anti-inflammatory, antihypertensive, and antioxidant effects [9, 10]. However, there is dearth of information on the possible mechanism of action of* M. oleifera* leaf extracts with respect to penile function and the ability of the leaves to protect the penis against Fe^2+^-induced lipid peroxidation. This study was designed to investigate the inhibitory effects of aqueous extract from* Moringa oleifera* leaves on key enzymes linked to ED (ACE and arginase) and its antioxidant potential.

## 2. Materials and Methods

### 2.1. Sample Collection

*Moringa oleifera* leaves were purchased from the Akure main market, Akure, Nigeria, and authenticated at the Department of Crop and Pest Management, Federal University of Technology, Akure, Nigeria. The* M. oleifera* leaves were air dried at room temperature and pulverized. Unless otherwise stated, all other chemicals and reagents used were of analytical grades and the water was glass distilled. A Jenway UV-visible spectrophotometer (Model 6305; Jenway, Barlo World Scientific, Dunmow, United Kingdom) was used to measure absorbance.

### 2.2. Phenolic Extraction

Powdered sample (5 g) was soaked in 100 mL of distilled water for about 24 h at 37°C. The mixture was filtered and centrifuged at 4000 rev/min for 10 min to obtain a clear supernatant which was used for subsequent analysis.

### 2.3. High Performance Liquid Chromatography-Diode Array Detector (HPLC) Analysis

Chromatographic analyses were carried out under gradient conditions using C_18_ column (4.6 mm × 150 mm) packed with 5 *μ*m diameter particles; the mobile phase was water containing 1% formic acid (A) and acetonitrile (B), and the composition gradient was 13% of B until 10 min and changed to obtain 20, 30, 50, 60, 70, 20, and 10% B at 20, 30, 40, 50, 60, 70, and 80 min, respectively [11].* Moringa oleifera* leaf extract and mobile phase were filtered through 0.45 *μ*m membrane filter (Millipore) and then degassed by ultrasonic bath prior to use; the extract was analyzed at a concentration of 20 mg/mL. The flow rate was 0.7 mL/min, injection volume was 40 *μ*L, and the wavelength was 254 nm for gallic acid, 280 nm for catechin and epicatechin, 325 nm for chlorogenic and ellagic acids, and 365 nm for quercetin, quercitrin, isoquercitrin, rutin, and kaempferol. Stock solutions of standards references were prepared in the HPLC mobile phase at a concentration range of 0.030–0.250 mg/mL for kaempferol, quercetin, quercitrin, isoquercitrin, rutin, catechin, and epicatechin and 0.050–0.450 mg/mL for ellagic, gallic, and chlorogenic acids. Chromatography peaks were confirmed by comparing their retention time with those of reference standards and by DAD spectra (200–500 nm).

### 2.4. Determination of Total Phenol Content

The total phenol content of the extract was determined as described by Singleton et al. [12]. Briefly, appropriate dilution of the extracts was oxidized with 2.5 mL 10% Folin-Ciocalteu's reagent (v/v) and neutralized by 2.0 mL of 7.5% NaCO_3_. The reaction mixture was incubated for 40 min at 45°C and the absorbance was measured at 765 nm in the spectrophotometer. Gallic acid was used as standard and the total phenol content was subsequently calculated as gallic acid equivalent.

### 2.5. Determination of Total Flavonoid Content

The total flavonoid content was determined using a slightly modified method [13]. Briefly, 0.5 mL of appropriate diluted extract was mixed with 0.5 mL of methanol, 50 *μ*L of 10% AlCl_3_, 50 *μ*L of 1 M potassium acetate, and 1.4 mL H_2_O. The mixture was incubated at room temperature for 30 min. Thereafter, the absorbance of the reaction mixture was subsequently measured at 415 nm. Quercetin was used as standard and the total flavonoid content was calculated as quercetin equivalent.

### 2.6. Nitric Oxide Scavenging Activity

Nitric oxide scavenging assay was performed using Griess reagent method [14]. Briefly, 0.3 mL of sodium nitroprusside (5 mM) was added to 1 mL of each of various concentrations of the extract. The tubes were then incubated at 25°C for 150 min. After 150 min, 0.5 mL of Griess reagent (equal volume of 1% sulphanilamide on 5% orthophosphoric acid and 0.01% naphthyl ethylenediamine in distilled water, used after 12 h of preparation) was added. The absorbance was measured at 546 nm.

### 2.7. Hydroxyl Radical Scavenging Ability

The method of Halliwell and Gutteridge [15] was used to determine the ability of the extract to prevent Fe^2+^/H_2_O_2_-induced decomposition of deoxyribose. The extract 0–100 *μ*L was added to a reaction mixture containing 120 *μ*L of 20 mg deoxyribose, 400 *μ*L of 0.1 M phosphate buffer, and 40 *μ*L of 500 *μ*M of Fe_2_SO_4_, and the volume was made up to 800 *μ*L with distilled water. The reaction mixture was incubated at 37°C for 30 min and the reaction was then stopped by the addition of 0.5 mL of 28% trichloroacetic acid. This was followed by addition of 0.4 mL of 0.6% thiobarbituric acid solution. The tubes were subsequently incubated in boiling water for 20 min. The absorbance was measured at 532 nm in a spectrophotometer.

### 2.8. Fe^2+^ Chelation Assay

The Fe^2+^-chelating ability of the extracts was determined using a slightly modified method [16, 17]. Freshly prepared 500 *μ*M FeSO_4_ (150 *μ*L) was added to a reaction mixture containing 168 *μ*L of 0.1 M Tris-HCl (pH 7.4), 218 *μ*L of saline (0.9%), and the extracts (0–25 *μ*L). The reaction mixture was incubated for 5 min, before the addition of 13 *μ*L 0.25% 1,10-phenanthroline (w/v). The absorbance was subsequently measured at 510 nm in a spectrophotometer. The Fe(II)-chelating ability was subsequently calculated.

### 2.9. Preparation of Penile Tissue Homogenate

The rats were decapitated under mild diethyl ether anesthesia and rapidly dissected. The penile tissues were removed and placed on ice and weighed. These tissues were subsequently homogenized in cold saline (1/10 w/v) with about 10-up-and-down strokes at approximately 1200 rev/min in a Teflon glass homogenizer. The homogenate was centrifuged for 10 min at 3000 ×g to yield a pellet that was discarded and a low-speed supernatant (S1) that was kept for lipid peroxidation assay.

### 2.10. Lipid Peroxidation and Thiobarbituric Acid Reactions Assay

Hundred micro liters (100 *μ*L) of penile homogenate supernatant was mixed with a mixture containing 30 *μ*L of 0.1 M Tris-HCl buffer (pH 7.4), extract (0–100 *μ*L), and 30 *μ*L of the prooxidant (250 *μ*M iron(II) sulphate). The volume was made up with 300 *μ*L of distilled water before incubation at 37°C for 2 h. The colour reaction was developed by adding 300 *μ*L of 8.1% SDS (sodium dodecyl sulphate) to the reaction mixture containing the homogenate, followed by the addition of 600 *μ*L of acetic acid/HCl (pH 3.4) and 600 *μ*L of 0.8% thiobarbituric acid (TBA). This mixture was incubated at 100°C for 1 h. The absorbance of thiobarbituric acid reactive species (TBARS) produced was measured at 532 nm. MDA (malondialdehyde) produced was expressed as % control [18, 19].

### 2.11. Angiotensin-I-Converting Enzyme (ACE) Inhibition Assay

The inhibition of ACE activity of the extract was determined according to the described method of Cushman and Cheung [20]. Different concentrations of the extract and 50 *μ*L of rabbit lungs ACE (EC 3.4.15.1) solution (4 mU/mL) were preincubated at 37°C for 15 min. Thereafter, enzymatic reaction was initiated by adding 150 *μ*L of 8.33 mM ACE substrate [hippuryl-l-histidyl-l-leucine (HHL)] in 125 mM of Tris-HCl buffer (pH 8.3) to the reaction mixture and incubated at 37°C for 30 min. The reaction was stopped by adding 250 *μ*L of 1 M HCl. The hippuric acid (Bz-Gly) produced by the reaction was extracted with 1.5 mL ethyl acetate. The mixture was then centrifuged to separate the ethyl acetate layer, after which the 1 mL of the ethyl acetate layer was transferred to a clean test tube and evaporated to dryness. The residue was redissolved in distilled water and its absorbance was measured at 228 nm. The average value from three determinations of each concentration was used to calculate the ACE inhibition at concentration between 1.25 and 6.30 *μ*g/mL which was used as control.

### 2.12. Arginase Inhibition Assay

Penile homogenates were prepared by homogenizing 10 g (w/v) of penile tissue in three volumes of homogenization cold buffer (phosphate buffer, pH 7.2). The homogenate was centrifuged for 20 min at 4000 r.p.m and the supernatant was used as the source of enzyme. Arginase activity was determined by the measurement of urea produced by the reaction of Ehrlich's reagent. The reaction mixture contained in final concentration 1.0 mM Tris-HCl buffer, pH 9.5, containing 1.0 mM MnCl 0.1 M arginine solution and 50 mM of the enzyme preparation in a final volume of 1.0 mL. The mixture was incubated for 10 min at 37°C. The reaction was terminated by the addition of 2.5 mL Ehrlich reagent (2.0 g of p-dimethylaminobenzaldehyde in 20 mL of concentrated hydrochloric acid and made up to 100 mL with distilled water). The optical density reading was taken after 20 min at 450 nm. The control experiment was performed without the test sample and the arginase inhibitory activity was expressed as percentage inhibition [21].

### 2.13. Data Analysis

The results of three replicates were pooled and expressed as mean ± standard deviation (S.D.). Student's *t*-test, one-way analysis of variance (ANOVA), and least significance difference (LSD) were carried out [22]. Significance was accepted at *p* ≤ 0.05. IC_50_ was determined using nonlinear regression analysis.

## 3. Results

### 3.1. Phenolic Profile

**Figure 1:**
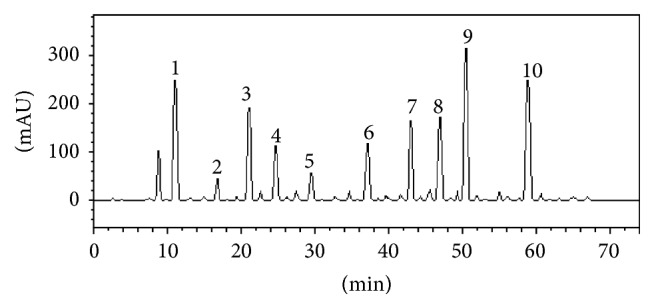
High performance liquid chromatography profile of* Moringa oleifera* leaf extract: gallic acid (peak 1), catechin (peak 2), chlorogenic acid (peak 3), ellagic acid (peak 4), epicatechin (peak 5), rutin (peak 6), quercitrin (peak 7), isoquercitrin (peak 8), quercetin (peak 9), and kaempferol (peak 10).

The HPLC-DAD analysis as presented in Table 1 and Figure 1 revealed the presence of phenolic compounds such as gallic acid (105.67 mg/g), catechin (20.19 mg/g), chlorogenic acid (79.31 mg/g), and ellagic acid (52.95 mg/g) and flavonoids such as quercetin (137.81 mg/g), quercitrin (74.9 mg/g), isoquercitrin (75.65 mg/g), kaempferol (106.75 mg/g), and rutin (60.38 mg/g). The results of the total phenol and flavonoid contents of the* M. oleifera* leaf extract are presented in Table 2. The total phenolic content reported as gallic acid equivalent was 15.2 mgGAE/100 g, while the total flavonoid content reported as quercetin equivalent was 3.1 mgQUE/100 g.

### 3.2. Radical Scavenging Ability

**Figure 2:**
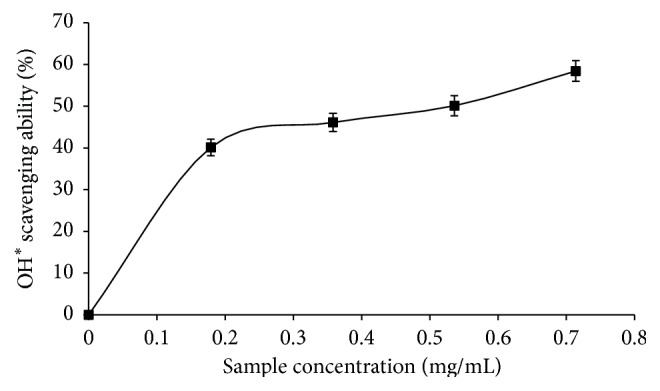
OH^*∗*^ scavenging ability of phenolic extract from* M. oleifera *leaves.

**Figure 3:**
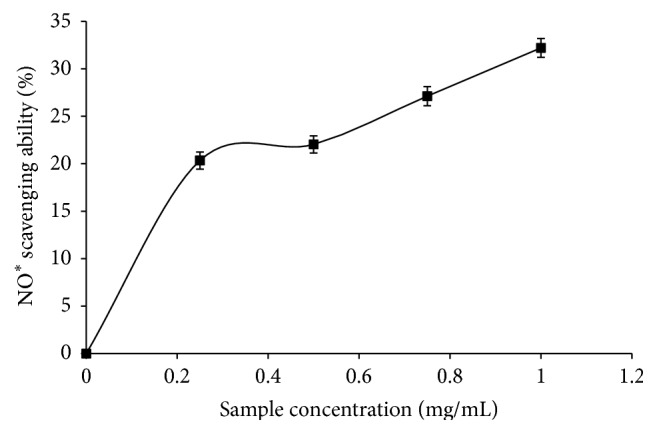
NO^*∗*^ scavenging ability of phenolic extract from* M. oleifera *leaves.

The phenolic extract dose-dependently scavenged OH^*∗*^ and NO^*∗*^ as shown in Figures 2 and 3, respectively. IC_50_ values are 0.52 mg/mL (OH^*∗*^) and 1.36 mg/mL (NO^*∗*^) (Table 3).

### 3.3. Fe^2+^-Chelating Ability

**Figure 4:**
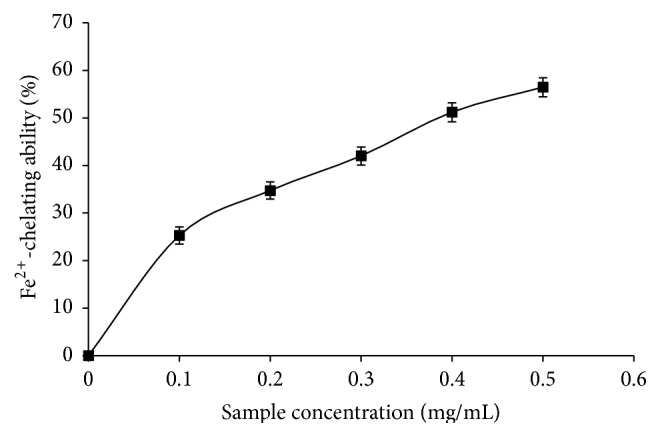
Fe^2+^-chelating ability of phenolic extract from* M. oleifera *leaves.

Figure 4 shows the Fe^2+^-chelating ability of the phenolic extract from* M. oleifera*. The extract was able to chelate Fe^2+^ in a dose-dependent manner with IC_50_ = 0.38 mg/mL as shown in Table 3.

### 3.4. Inhibition of Malondialdehyde Production

**Figure 5:**
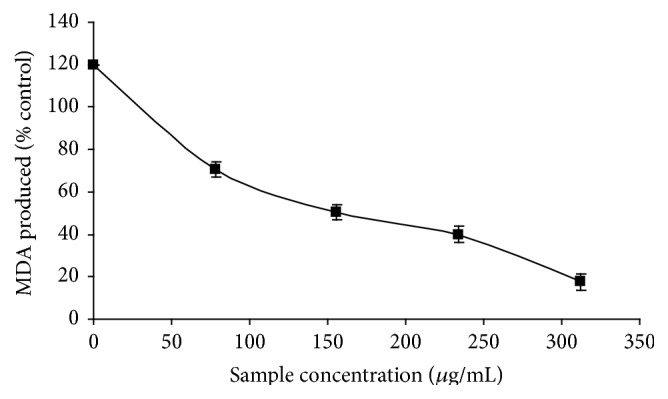
Inhibition of Fe^2+^-induced lipid peroxidation in rat penile tissue homogenate by phenolic extract from* M. oleifera *leaves.

The incubation of penile tissue homogenates in the presence of Fe^2+^ caused a significant increase (*p* < 0.05) in the malondialdehyde (MDA) content (120.58%) as shown in Figure 5. However, the phenolic extract from* M. oleifera* leaves inhibited MDA levels in a dose-dependent manner with IC_50_ value of 194.23 *μ*g/mL (Table 3).

### 3.5. Effects of Phenolic Extract from* M. oleifera* Leaves on ACE and Arginase Activities

**Figure 6:**
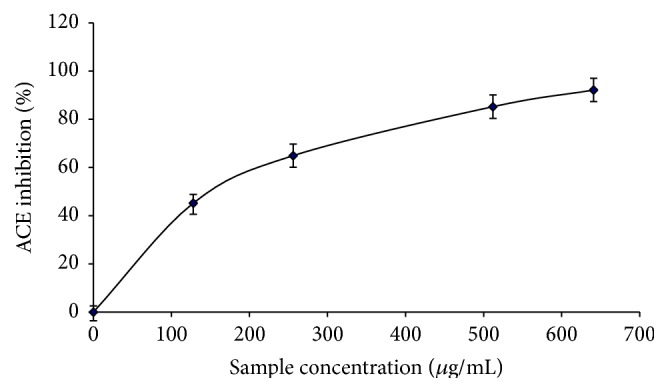
ACE inhibitory ability of phenolic extract from* M. oleifera *leaves.

**Figure 7:**
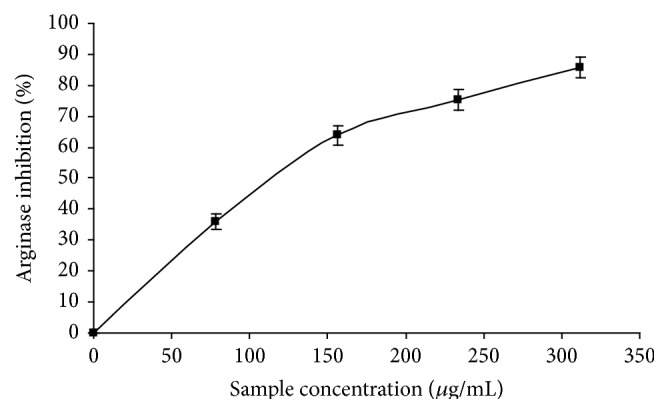
Arginase inhibitory ability of* Moringa oleifera *leaf extract.

The interaction of phenolic extract with ACE as shown in Figure 6 revealed that the phenolic extract from* M. oleifera* leaves inhibited ACE activity* in vitro* in a dose-dependent pattern with IC_50_ of 303.03 *μ*g/mL. Furthermore, the result in Figure 7 revealed that the phenolic extract inhibited arginase activity in a dose-dependent manner with IC_50_ = 159.59 *μ*g/mL (Table 3).

## 4. Discussion

*M. oleifera *is known to have various medicinal benefits and these have been attributed to its phytochemicals such as phenolic compounds [10, 23]. These phytochemicals are capable of causing definite physiological actions in human body. Reports have revealed that phenolic contents of medicinal plants are related to their antioxidant capacity [24]. The result obtained in this study shows that the phenolic extract from* M. oleifera leaves *exhibited hydroxyl (OH) and nitric oxide (NO) radicals scavenging abilities. Hydroxyl radical (OH) is mainly generated in biological systems from superoxide anion and hydrogen peroxide by the Haber-Weiss reaction or from hydrogen peroxide via the Fenton reaction [25]. Hydroxyl radical (OH^*∗*^) is highly reactive, energetic, short-lived, and very toxic to cells [26]. Studies have revealed that reactive species such as hydroxyl radicals (OH^*∗*^) play an important role in the diabetes-related ED [27, 28]. The OH^*∗*^ scavenging ability of the* M. oleifera* leaf extract could be attributed to the presence of polyphenols which are capable of donating hydrogen atoms to OH radicals, thus inhibiting the oxidation process [29]. Nitric oxide radical (NO^*∗*^), generated by the inducible form of nitric oxide synthase (NOS) in response to inflammation, mediates many cytotoxic and pathological processes and could contribute in part to the formation of plaque in penile tissues [30]. Although NO is required for the initiation of erection by mediation of the relaxation of corpus cavernosum smooth muscles and penile tissues, it can also combine with superoxide (O^2−^) to form peroxynitrite. The interaction between NO and reactive oxygen species (ROS) is central to the pathophysiology of ED [31]. Peroxynitrite is cytotoxic and contributes to lipid peroxidation and nitration which leads to the formation of malondialdehyde and hydroperoxides [32]. Although peroxynitrite causes smooth-muscle relaxation, it also increases the incidence of apoptosis in the endothelium and decreases the synthesis and bioavailability of NO [33]. Therefore, the NO^*∗*^ scavenging ability of the* M. oleifera* leaf extract as revealed in this study could be beneficial in the management of erectile dysfunction. Hence, augmenting the body's antioxidant status could be a practical approach by which oxidative stress-induced erectile dysfunction can be managed.

The disruption of iron balance in the body system can bring about iron overload which is associated with oxidative stress-induced ED [34]. Iron overload increases the formation of reactive oxygen species (ROS) which induces the initiation of lipid peroxidation [35]. Iron II (Fe^2+^) reacts with H_2_O_2_ in the Fenton reaction to produce the highly reactive hydroxyl radical, which can damage proteins, lipids, and nucleic acids. Our findings revealed that phenolic extract from* M. oleifera* leaves was able to chelate Fe^2+^ in a dose-dependent manner. This chelating ability might be due to the presence of some phytochemicals such as polyphenols. Phenolic compounds can form a complex with iron thereby aiding its excretion from the body. Fe^2+^-chelating ability of* M. oleifera* leaf extract could therefore be beneficial in the management/prevention of erectile dysfunction [36]. Moreover, Akomolafe et al. [37] suggested phenolic compounds such as gallic acid, chlorogenic acid, catechin, kaempferol, quercetin, and quercitrin are capable of interfering with iron metabolism thereby chelating the metallic ion.

Superoxide anions and other reactive oxygen species have been shown to be a major contributor to the pathogenesis of erectile dysfunction via the initiation of lipid peroxidation [38, 39]. Increase in malondialdehyde (MDA) content when rat penile tissue homogenate was incubated in the presence of Fe^2+^ could be through the breakdown of hydrogen peroxide to generate OH^*∗*^ [40]. Fe^2+^-induced lipid peroxidation in the cell membrane induces oxidative stress, reduces antioxidant enzymes, and causes injuries to the penile tissues [41]. According to Jia et al. [42] oxidative damage can occur in the DNA via peroxidative breakdown of membrane polyunsaturated fatty acids. DNA damage affects homeostasis of various cells leading to cell death [43]. However, the inhibition of MDA production caused by the extract from* M. oleifera* leaves could be attributed to the ability of the extract to chelate Fe^2+^ and scavenged radicals thus preventing the initiation of oxidative damage [39].

Some experimental investigations have revealed that increase in blood pressure may be associated with erectile dysfunction [44]. Therefore inhibition of angiotensin-I-converting enzyme which catalyses the conversion of angiotensin I to angiotensin II has marked effects on lowering the blood pressure thereby activating the release of NO which improves erectile function. Furthermore, the inhibition of ACE activates bradykinin which has been implicated in erectile function [5, 44]. ACE inhibitors have a valuable role to play in patients who have hypertension and erectile dysfunction. The observed inhibitory effects of the phenolic extract from* M. oleifera* leaves on ACE activity could be linked to the phenolic constituents and this could contribute to erectile function. Previous report has shown that phenolics can interact with disulphide bridges present on the surface of the enzyme thereby modifying the structure and reducing its activity [45].

Decrease in NO bioavailability due to endothelial dysfunction or nerve injury is a major predisposing factor for erectile dysfunction [45]. An increased level of arginase is common in patients with ED due to decrease in nitric oxide synthase (NOS) activity and impairment in the biosynthesis of nitric oxide (NO) via the NO-cGMP pathway [46]. Thus, dose-dependent inhibition of arginase activity by the* M. oleifera* leaf extract could be of immense importance in the management of ED as this would increase genital blood flow during sexual arousal. The inhibitory properties of the phenolic extract from* M. oleifera *leaves could be attributed to the phenolic components. Polyphenols have been reported to possess inhibitory effects on arginase activity [47, 48]. Furthermore, flavonoids such as catechin, epicatechin, and quercetin and its derivatives (quercitrin and isoquercitrin) have shown to be strong inhibitors of arginase activity and could be linked to the formation of hydrogen bond and hydrophobic interactions between these polyphenolic compounds and the hydrophobic active site of the enzyme [47, 48].

## 5. Conclusion

The antioxidant properties and ACE and arginase inhibitory effects of phenolic extract from* M. oleifera* leaves suggest that this plant has a therapeutic potential in the management of erectile dysfunction. These findings also revealed the possible mechanism of action of the* M. oleifera* leaf in the management/treatment of ED. However, this health promoting effect is suggested to be a function of its phenolic and flavonoid contents.

## Figures and Tables

**Table 1:** Chemical composition of *Moringa oleifera* leaf extract.

Component	mg/g	%
Gallic acid	105.67 ± 0.01	10.56
Catechin	20.19 ± 0.03	2.01
Chlorogenic acid	79.31 ± 0.02	7.93
Ellagic acid	52.95 ± 0.02	5.29
Epicatechin	29.73 ± 0.01	2.97
Rutin	60.38 ± 0.02	6.03
Quercitrin	74.90 ± 0.01	7.49
Isoquercitrin	75.65 ± 0.02	7.56
Quercetin	137.81 ± 0.01	13.78
Kaempferol	106.75 ± 0.03	10.67

Values represent means ± standard deviation of triplicate readings.

**Table 2:** The total phenolic and flavonoid content of *M. oleifera* leaf extract (mg/100 g).

Parameter (unit)	Value
Total phenol (gallic acid equivalent) (mg/100 g)	15.2 ± 1.6
Total flavonoid (quercetin equivalent) (mg/100 g)	3.1 ± 0.1

Values represent means ± standard deviation of triplicate readings.

**Table 3:** IC_50_ values of NO^*∗*^ and OH^*∗*^ scavenging and Fe^2+^-chelating abilities and inhibition of ACE and arginase activities and Fe^2+^-induced lipid peroxidation in rat's penis homogenates by phenolic extract from *M. oleifera *leaves.

Parameter	IC_50_
NO scavenging ability (mg/mL)	1.36 ± 0.09
OH scavenging ability (mg/mL)	0.52 ± 0.07
Fe^2+^-chelating ability (mg/mL)	0.38 ± 0.08
Inhibition of Fe^2+^-induced lipid peroxidation (*µ*g/mL)	194.23 ± 2.31
Inhibition ACE activity (*µ*g/mL)	303.03 ± 3.45
Inhibition of arginase activity (*µ*g/mL)	159.59 ± 2.14

Values represent means ± standard deviation of triplicate readings.
